# Psychiatric comorbidity in functional tics: a scoping review

**DOI:** 10.1186/s12888-026-07932-2

**Published:** 2026-03-07

**Authors:** Michael N. Nemeh, Peggy Tahir, Matthew E. Hirschtritt, Raj K. Kalapatapu

**Affiliations:** 1 Department of Psychiatry and Behavioral Sciences, University of California, San Francisco, 675 18th St, San Francisco, CA 94107 USA; 2 UCSF Library, University of California, San Francisco, USA; 3 Division of Research, Kaiser Permanente Northern California, Oakland, CA USA; 4 Department of Psychiatry, Kaiser Permanente Oakland Medical Center, Oakland, CA USA

**Keywords:** Functional tics, Functional neurologic disorder, Comorbidity, Depression, Anxiety

## Abstract

**Background:**

There has been a dramatic rise in the prevalence of functional tics since the COVID-19 pandemic. While the prevalence of various comorbidities has been well defined in primary tic disorders such as Tourette Syndrome, less is known about this topic in patients with functional tics. Therefore, the purpose of this scoping review is to characterize what is known about the prevalence of psychiatric and neurologic comorbidities in patients with functional tics and identify gaps that persist in this literature.

**Methods:**

A comprehensive search across multiple databases was used for data collection. We included studies that provided original data on the presence of psychiatric comorbidities in patients with a diagnosis of functional tics. Study screening progress was documented in a Preferred Reporting Items for Systematic Reviews and Meta-Analyses flow chart. A total of 150 titles and abstracts were screened, 78 full texts were evaluated for eligibility, and 31 studies were included.

**Results:**

The included studies identified epidemiological data and common psychiatric and neurologic comorbidities in patients with functional tics. Most of the studies reviewed were published after 2020, highlighting the recent uptick in incidence and prevalence of functional tics. Depression and anxiety, followed by attention deficit hyperactivity disorder and obsessive-compulsive disorder, were among the most commonly identified comorbidities.

**Conclusions:**

Overall, further research on the prevalence of comorbidities in patients with functional tics is needed to inform clinicians’ differential diagnosis and integrated treatment planning. Depression and anxiety are common comorbidities in patients with functional tics and may be underrecognized and underreported. The prevalence of all comorbidities appears to have increased since the COVID-19 pandemic. Future research should further quantitatively define the comorbidity profile in functional tics.

**Clinical trial number:**

Not applicable.

**Supplementary Information:**

The online version contains supplementary material available at 10.1186/s12888-026-07932-2.

## Introduction

## Background

Tics, defined as “sudden, rapid, recurrent, nonrhythmic motor movement or vocalizations” in the DSM-5, can be a source of significant morbidity for patients, including social embarrassment and physical discomfort [1, 2]. Tics can occur as part of a primary tic disorder such as Tourette Syndrome (TS), and they can also occur within the spectrum of functional neurologic disorder (FND). Functional tics (FT), often referred to as functional tic-like behaviors (FTLB), can be differentiated from tics in primary tic disorders in that they are more common in females, have later onset, have significant variability, lack typical rostro-caudal progression, and are sudden-onset [2]. Differentiating these two conditions involving tics is critical for treatment planning. Diagnosis of FT is challenging and should not be based on phenomenology alone, as both FT and neurodevelopmental tics, such as those seen in TS, can share features such as distractibility and inconsistency [3]. Instead, consideration of comorbidity in a patient presenting with tics can inform differential diagnosis. Moreover, since the start of the COVID-19 pandemic, there has been a drastic rise in the number of patients diagnosed with FT, underscoring the need to improve characterization of this condition and its comorbidity profile [3]. Organizations such as the European Society for the Study of Tourette Syndrome (ESSTS) have put forward criteria for establishing the diagnosis of functional tics, which in part includes assessment of the patient’s comorbidity profile [4].

A consistent theme across both primary tic disorders and FT is the high prevalence of psychiatric comorbidities. The prevalence of comorbidities in Tourette Syndrome is well established, with obsessive-compulsive disorder/obsessive-compulsive behavior (OCD/OCB) and attention deficit hyperactivity disorder (ADHD) being the most common [5]. In contrast, the prevalence of comorbidities in FT is less well-defined. It is thought that in FT there may be a relatively higher prevalence of comorbid mood disorders, particularly anxiety, and trauma-related conditions [4]. Defining the prevalence of various comorbidities in FT is clinically meaningful for several reasons; accurate characterization of the comorbidity pattern in FT will inform differential diagnosis, and understanding associations between FT and other psychiatric disorders will be essential for developing integrated treatment plans which would likely improve outcomes for these patients [6].

### Importance

While systematic reviews are used to synthesize and assess research related to a specific question or questions, scoping reviews can be used to identify gaps in the literature by exploring broader questions and mapping existing literature on a topic [7]. At present, there has been no comprehensive synthesis mapping the available evidence on psychiatric comorbidities in functional tics. As a result, the published literature on this topic is limited and fragmented; therefore, a scoping review is likely to capture the existing literature that addresses this topic better than a more restrictive and structured systematic review.

### Goals of this investigation

This scoping review aims to examine the prevalence of various psychiatric and neurologic comorbidities in FT and the gaps in our understanding of this topic.

## Methods

### Study design and registration

The current scoping review followed guidelines described in the Preferred Reporting Items for Systematic Reviews and Meta-Analyses Extension for Scoping Reviews (PRISMA-ScR) [8]. Given that this was designed as a scoping review in which we modified and expanded our search strategy based on our initial search results, we did not preregister this scoping review on a publicly available database. This scoping review has no source of funding and is solely the responsibility of the authors.

### Search strategy

We searched PubMed, Web of Science, Embase, and PsycINFO databases to find articles for our review. Searches were developed to query the literature on the topics of functional tics, with multiple synonyms developed for each concept to create sensitive and broad searches. The full search strategies for each database are included as Table 1 (Table 1, see Additional File 1). Searches were conducted on 7/29/2025. The only limits imposed were limiting the Embase results to articles, articles in press, and reviews, and limiting the PsycINFO searches to peer-reviewed articles. Our gray literature searches included reviewing the references in the articles selected for data extraction and conducting Google searches for white papers, statistics, and other relevant background information.

### Selection of studies

To be included in our study, the identified papers provided original comorbidity data in human patients with a primary diagnosis of functional tics. We included papers studying any age range, any location, any clinical setting, and any time-period. Excluded from our study were papers that examined non-human subjects, non-peer-reviewed articles, review articles, articles where functional tics are not the primary diagnosis or there was diagnostic uncertainty, and articles that are not in English if no translation services were available. No restrictions on publication year were set.

**Fig. 1:**
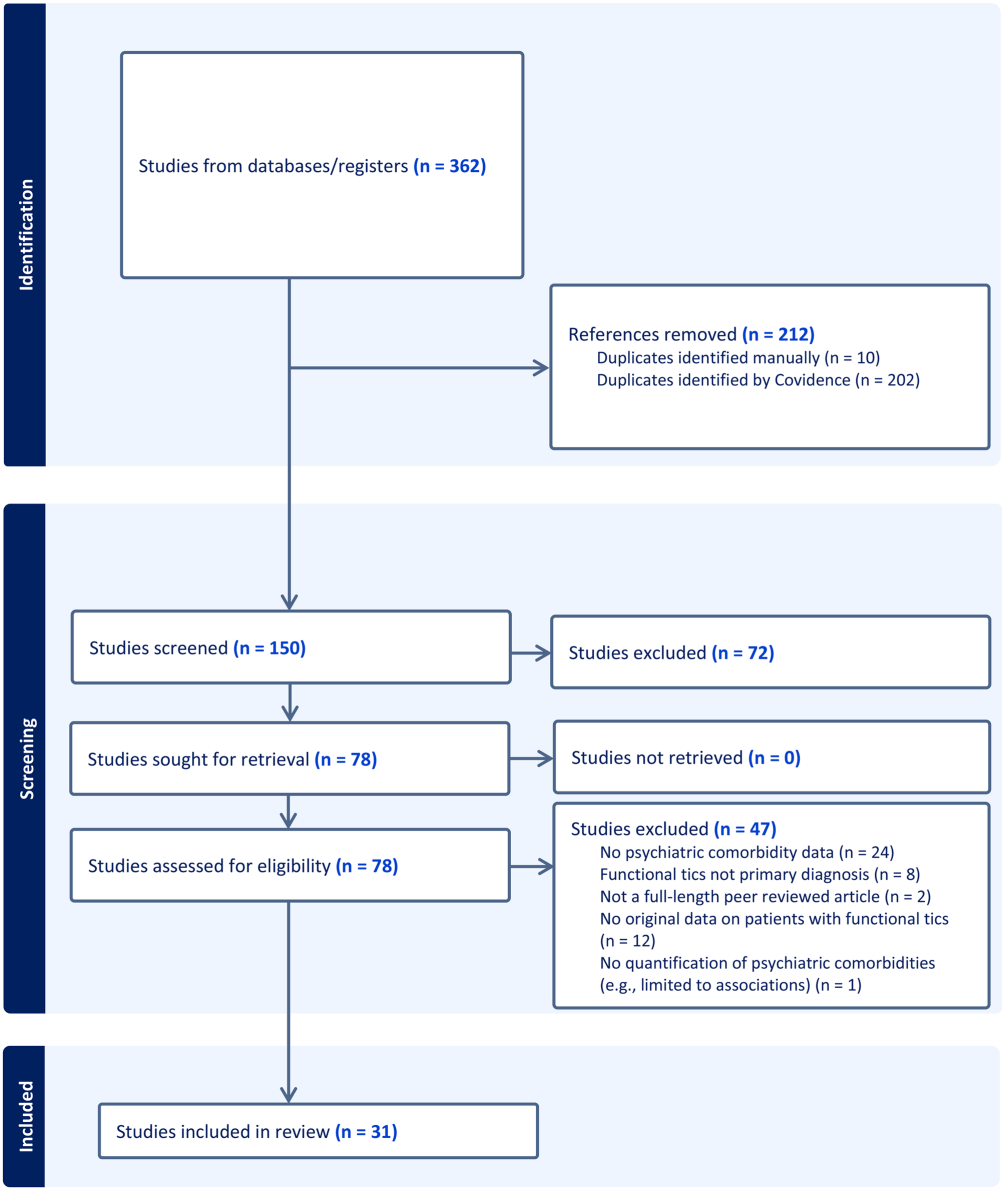
Preferred reporting items for systematic reviews and meta-analyses (PRISMA) flow diagram

Study screening progress was documented in a PRISMA flow chart (Fig. 1) [9]. After removing duplicates, our search strategy yielded 150 publications. Publications were then divided and screened by three reviewers (MN, MH, RK) using Covidence systematic review software to determine if they met criteria for full-text review [10]. Sixty-seven were eliminated because of irrelevance to the topic. Full-text screening of 78 articles was completed independently by the three reviewers to determine eligibility for inclusion. Of the 78 full texts reviewed, 47 did not meet the inclusion criteria because they did not provide original data on the prevalence of comorbidities in patients with a confirmed diagnosis of functional tics. The three reviewers assessed and summarized findings from the final 31 articles. Any disagreements in the full-text review were resolved collectively by all four authors through consultation and detailed examination of the study. All authors met regularly to discuss any questions regarding the articles to ensure consistency in decision-making.

### Data extraction and synthesis

Data were initially extracted by the research librarian (PT) and then charted by the other three authors (MN, MH, RK) into a standardized electronic form, including information about study design, study population, study location, and prevalence of comorbidities. We performed a narrative synthesis because of heterogeneous study designs in the context of a scoping review. No single summary measure was applicable across all studies.

## Results

### Characteristics of included studies

A total of 31 studies published between 2015 and 2025 met the inclusion criteria (Fig. 1). Table 2 describes characteristics of studies that met the inclusion criteria (Table 2, see Additional File 2). Studies were conducted across diverse geographic regions, as 8 were from the UK, 7 were from the United States, 6 were from Germany, 4 were from Canada, 2 were from Denmark, 2 were from Australia, and 2 were from multiple countries. All papers reported quantitative data regarding the prevalence of comorbid psychiatric conditions among individuals with FT. Study design was heterogeneous. For example, of the 31 studies, 3 were prospective cohort studies and the remaining (*N* = 28) were retrospective, including 16 retrospective cohort studies, 7 cross-sectional studies, and 5 case series. Of the included studies, 23 had greater than 50% female participants. This trend was reversed when considering data from Germany, where a majority of participants were male. Similarly, all 4 studies published before 2020 had majority male participants. Race and/or ethnicity of the individuals with FT were reported in one study that had 100% white participants.

The included studies varied in terms of method of diagnosing functional tics, method of diagnosing comorbid conditions, study conditions, and temporal relationship to the COVID-19 pandemic, all of which may contribute to differences in prevalence of comorbidities across studies. In terms of establishing the diagnosis of functional tics, a range of approaches were taken including expert clinical judgment, structured diagnostic methods (e.g. ESSTS criteria), and retrospective chart review. Similarly, comorbid psychiatric conditions were diagnosed through diverse methods, such as standardized instruments (e.g. Patient Health Questionnaire-9), clinician assessment, or caregiver report. Study conditions ranged from routine primary care environments to specialized tic-focused clinics, with some studies drawing from national registries or multi-center collaborations. Temporal context also varied across studies. A minority (*N* = 11) included data collected before the COVID-19 pandemic and only 4 of those studies were published before 2020; the remaining studies (*N* = 20) exclusively reported data that was collected in 2020 or later.

### Prevalence of reported comorbidities

Table 3 describes the prevalence of comorbidities across studies (Table 3, see Additional Table 3). Depression and anxiety were among the most common comorbidities. Prevalence of depression comorbid with functional tics ranged from 10% to 100%, with several studies reporting a prevalence of ~ 30–45% [11, 12]. The prevalence of comorbid anxiety was consistently high as well, often greater than 60% [13–15]. Although this was not always the case, studies that used structured interviews to diagnose comorbidities tended to report a higher prevalence of depression and anxiety compared to studies that relied on chart review or caregiver report. For example, Firestone et al., using standardized questionnaires to diagnose comorbidities, found that 100% of participants had both depression and anxiety, whereas Buts et al., using parental report, reported lower prevalence of comorbid depression and anxiety at 24% and 68% respectively. The prevalence of ADHD was variable, from 10% to greater than 50% [16, 17]. Studies that used structured interviews tended to report lower prevalence of ADHD versus those that used parental report or chart review. There was also a wide range of reported prevalence of OCD/OCB, with some studies describing greater than 70% and others much lower [18]. Reported prevalence of comorbid ASD was generally around 10–25%. The prevalence of all comorbidities appeared to increase peri- and post-pandemic.

In terms of less commonly reported psychiatric comorbidities, PTSD or prior history of trauma as well as borderline personality disorder were not infrequently reported as comorbidities in patients with functional tics. Other comorbidities identified include bipolar disorder, agoraphobia, panic disorder, substance use disorder, psychosis, anorexia, social anxiety disorder, separation anxiety disorder, intellectual disability, sleeping problems, suicidal ideation or attempts, alexithymia, self-harm, pica, specific phobia, conduct disorder, oppositional defiant disorder, and dissociation.

Functional neurological disorder was a commonly reported comorbidity, with several different subtypes of FND noted including functional seizures and functional weakness [13, 19]. Other reported neurologic comorbidities included gait abnormalities, headaches or migraines, seizures, and restless leg syndrome.

## Discussion

In our broad search of the current literature, we identified 31 studies that reported on the prevalence of comorbid psychiatric conditions in patients with FT. This paper lays the foundations for further research that should quantitatively and systematically synthesize data on the prevalence of comorbid conditions and compare that data to other conditions such as Tourette Syndrome. Characterization of the comorbidity profile in functional tics is critical for differentiating FT from neurodevelopmental tics such as TS and for guiding comprehensive treatment planning for patients who have been diagnosed with FT.

While it is challenging to draw strong conclusions in the context of a scoping review, the current evidence available suggests major themes within the existing literature on comorbidity in patients with functional tics. First, psychiatric comorbidity is ubiquitous in FT, with anxiety and depression most consistently noted. The prevalence of comorbid depression and anxiety appeared to be higher in studies that used validated measures to diagnose comorbidities, indicating that affective disorders may be underrecognized and underreported in patients with FT. Future studies should adopt standardized, validated diagnostic tools when investigating the prevalence of comorbidities in patients with FT so that the comorbidity profile can be accurately characterized.

Along with mood disorders, ADHD and OCD/OCB are also common; however, prevalence varies widely, possibly reflecting heterogeneity of diagnostic methods across the studies included (e.g. chart review vs. structured interview) or the lower prevalence of these comorbidities in patients with FT compared to TS. Trauma-related and personality disorders appear more in adult samples, possibly suggesting age-related differences in comorbidity profiles. Clinicians caring for patients with FT should be mindful that these patients may present with additional FND symptoms. Of note, most studies had greater than 50% of females among their participants. This trend is reversed in Germany, where most studies reported a preponderance of males. This may be because the most-viewed German tic social media influencer, Gewitter Im Kopf, is a man [20]. It is also unclear whether the finding that all 4 studies published before 2020 included a majority of male participants represents bias in sampling or FT diagnosis prior to 2020 versus an asymmetric increase in the prevalence of FT in females peri- and post-pandemic. Furthermore, this scoping review highlights the elevated prevalence of ASD in patients with FT compared to the general population, which concords with recent findings on the topic [21–23]. Finally, some studies noted the presence of a stressor, particularly the COVID-19 pandemic, prior to the development of FT symptoms; moreover, exposure to tics on social media prior to the onset of symptoms was frequently noted.

Elucidating the comorbidity profile in FT will enhance clinical practice by guiding differential diagnosis and integrated treatment planning. For example, in Tourette Syndrome, ADHD and OCD/OCB are the most common comorbidities; on the other hand, depression and anxiety may be less common in TS compared to FT [5]. If this is the case, as part of their comprehensive assessment, clinicians caring for patients with FT should have a higher index of suspicion for comorbid anxiety and depression compared to patients with TS. Clinicians should also bear in mind, based on our findings, that the prevalence of comorbidities in patients with FT may have increased since the pandemic. Given the limitations of scoping reviews, future work, including systematic reviews and meta-analyses, should aim to establish the comorbidity profile in FT conclusively and quantitatively.

To our knowledge, this is the first scoping review mapping the prevalence of psychiatric and neurologic comorbidities in patients with FT. Strengths of this article include a comprehensive search strategy across multiple databases, ideally ensuring the inclusion of all available data. Moreover, the inclusion of global data enhances generalizability. This study is also timely and relevant given the finding that the number of studies documenting comorbidity in FT has surged since the pandemic. Finally, beyond offering some practical guidance for clinicians, this review sets the stage for future investigation on FT, including meta-analyses.

This scoping review is not without limitations. Among the 31 articles identified, there was significant heterogeneity in the study designs. This led to more breadth, but less in-depth conclusions on the topic. Heterogeneity of study design in the context of a scoping review also precludes quantitative synthesis of comorbidity prevalence. Diagnostic method for functional tics varied across study design, and reports of comorbidities are likely more valid in studies that used more stringent diagnostic methods, such as ESSTS criteria, to establish the diagnosis of FT. Diagnostic method for comorbidities also varied across studies, with some using structured interviews and others relying on chart review or parental report of comorbidities. Most studies did not use validated measures to diagnose comorbidities, limiting the quality and reliability of the data. While we tried to use a variety of search terms, there is still the potential to have missed studies that did not fit with our terminology.

While this scoping review identifies the prevalence of comorbidities in patients with functional tics, there is still much to be learned on this topic. The scarcity of the literature on this topic demonstrates a gap in current mental health research and elucidates the need for more exploration on the experiences of patients with functional tics in order to better inform clinical practice.

## Supplementary Information

Below is the link to the electronic supplementary material.

Supplementary Material 1

Supplementary Material 2

Supplementary Material 3

## Data Availability

All data generated or analyzed during this study are included in this published article and additional files.

## Abbreviations

ADHD: Attention Deficit Hyperactivity Disorder
ASD: Autism Spectrum Disorder
ESSTS: European Society for the Study of Tourette Syndrome
FND: Functional Neurological Disorder
FT: Functional tics
FTLB: Functional tic-like behaviors
TS: Tourette Syndrome
OCD: Obsessive-Compulsive Disorder
OCB: Obsessive-Compulsive Behaviors
PRISMA-ScR: Preferred Reporting Items for Systematic reviews and Meta-Analyses extension for Scoping Reviews
PTSD: Post-Traumatic Stress Disorder
