# Supplementary material for: Psychiatric comorbidity in functional tics: a scoping review
Source: BMC Psychiatry. 2026 Mar 7;26:314. doi: 10.1186/s12888-026-07932-2 (PMC13081296; doi:10.1186/s12888-026-07932-2)
Supplement: Supplementary file 1 — Supplementary Material 1 [file 12888_2026_7932_MOESM1_ESM.docx]

**Additional File 1**

**Table 1. Database Search Strategy**

| DATABASE | SEARCH STRATEGY |
| --- | --- |
| PubMed | (“functional tic” OR “functional tics” OR “tic-like behavior” OR “tic-like behaviors” OR FTLBs OR “Tourette-like behavior” OR “tourette-like behaviors” OR ("Tics"[Mesh] OR "Tourette Syndrome"[Mesh]) AND (“functional tic” OR “functional tics” OR “tic-like behavior” OR “tic-like behaviors” OR FTLBs OR “Tourette-like behavior” OR “tourette-like behaviors”)) |
| Web of Science | (“functional tic” OR “functional tics” OR “tic-like behavior” OR “tic-like behaviors” OR FTLBs OR “Tourette-like behavior” OR “tourette-like behaviors”) |
| PsycINFO | (“functional tic” OR “functional tics” OR “tic-like behavior” OR “tic-like behaviors” OR FTLBs OR “Tourette-like behavior” OR “tourette-like behaviors”) |
| Embase | ('functional tic' OR 'functional tics' OR 'tic-like behavior' OR 'tic-like behaviors' OR 'ftlbs' OR 'tourette-like behavior' OR 'tourette-like behaviors' OR 'tic'/exp OR 'gilles de la tourette syndrome'/exp) AND ('functional tic' OR 'functional tics' OR 'tic-like behavior' OR 'tic-like behaviors' OR 'ftlbs' OR 'tourette-like behavior' OR 'tourette-like behaviors') |

**Table 1. Database search strategy**
