# Supplementary material for: Psychiatric comorbidity in functional tics: a scoping review
Source: BMC Psychiatry. 2026 Mar 7;26:314. doi: 10.1186/s12888-026-07932-2 (PMC13081296; doi:10.1186/s12888-026-07932-2)
Supplement: Supplementary file 2 — Supplementary Material 2 [file 12888_2026_7932_MOESM2_ESM.docx]

**Additional File 2**

**Table 2. Description of Studies that Met Inclusion Criteria.**

| **Author and Year** | **Study Design** | **Study Location** | **Data Collection Period** | **# Participants** | **Mean Age of Participants (Range)** | **Sex ratio** | **Method of diagnosis of secondary tic disorder** | **Method of diagnosis of comorbidities** | **Notes** |
| --- | --- | --- | --- | --- | --- | --- | --- | --- | --- |
| Anderson et al., 2023 (1) | Retrospective review comparing pediatric patients with FTLB and organic tics in terms of demographics and tic phenomenology | Copenhagen, Denmark | May 2020 to April 2022 | 53 | 13.7 (6-19.8) at onset  14.9 (11-20.2) at assessment | 94.3% F | Assessment by senior child neurologists, diagnostic uncertainty resolved with multidisciplinary conference | Chart review or guardians stating diagnosis during medical interview | 56.6% of FTLB patients confirmed exposure to tics on social media |
| Armstrong-Javors et al., 2024 (2) | Retrospective, cross-sectional review comparing patients with FT who presented to clinic before and during COVID-19 pandemic | Boston, USA | Pre-pandemic group: May 2018 to March 2020; During-pandemic group: March 2020 to January 2022 | 19 | 14.3 (10-26) at onset | 95% F assigned at birth  37% sexual orientation or gender minority | Not specified. Authors collected data on all patients who presented to clinic with FT ICD diagnosis and excluded those whose presentations were inconsistent with FT on further expert review | Chart review |  |
| Baizabal-Carvallo et al., 2023 (3) | Video-recordings of patients and medical record review with tics were assessed by experts to compare features of Tourette syndrome and STD | Houston, USA | 3 year period, not further specified | 21 | 31.6 at onset  35.7 at assessment | 47.7% F | Video-recordings of patients and medical record review with tics were assessed by experts | NR |  |
| Berg et al., 2024 (4) | Cross-sectional comparison of features in patients with FTLB, TS, and neurotypical participants aged 11-25 | Calgary, Canada | October 2020 to June 2022 | 35 | 17.5 at assessment | 91.4% assigned F at birth  42.8% gender minority | Diagnosis confirmed by neurologists based on ESSTS criteria | Clinical interview using MINI and MINI-KID along with retrospective chart review |  |
| Burn et al., 2025 (5) | Qualitative studies based on semi-structured interviews with 9 females aged 12-18 with a formal diagnosis of FT | London, UK | 2021 to 2023 | 9 | 15.2 (12-18) | 100% F | NR | Semi-structured interviews |  |
| Buts et al., 2022 (6) | Retrospective chart review of adolescents diagnosed with FTLB | London, UK; Calgary, Canada | November 2020 to April 2021 | 34 | 13.7 at onset | 94% F | Chart review, sudden onset being a primary criterion | Parent/guardian report of previously diagnosed comorbidities |  |
| Cavanna et al., 2025 (7) | Retrospective review comparing patients with TS who developed FTLB during pandemic with patients who only had TS | Birmingham, UK | January 2023 to March 2025 | 63 | 22.4 (12-60) at onset  24 (13-40) at assessment | 69.8% F | Assessment by behavioral neurologist using semi-structured interview (National Hospital Interview Schedule for Tourette syndrome) and ESSTS | NR | There is likely some overlap in the patient populations in each Cavanna et al. study |
| Cavanna et al., 2023 (8) | Retrospective review comparing patients with functional tics but no neurodevelopmental tics or other functional symptoms to patients with other functional symptoms | Birmingham, UK | April 2020 to April 2023 | 66 | 21.1 (11-61) at onset  23.1 (13-63) at assessment | 71.2% F | Assessment by behavioral neurologist using semi-structured interview (National Hospital Interview Schedule for Tourette syndrome) and ESSTS | NR |  |
| Cavanna et al., 2023 (9) | Retrospective review comparing patients who developed functional tics during the pandemic to age and gender matched patients with Tourette syndrome | Birmingham, UK | April 2020 to March 2023 | 83 | 21.2 (11-6) at onset  23.2 (13-63) at assessment | 71.1% F | Assessment by behavioral neurologist using semi-structured interview (National Hospital Interview Schedule for Tourette syndrome) and ESSTS | NR |  |
| Cavanna et al., 2023 (10) | Cross-sectional case series on patients who developed functional tics during COVID-19 pandemic | Birmingham, UK | April 2020 to March 2023 | 105 | 21.4 (11-61) at onset  23.2 (13-63) at assessment | 72.4% F | Assessment by behavioral neurologist using semi-structured interview (National Hospital Interview Schedule for Tourette syndrome) and ESSTS | NR |  |
| Demartini et al., 2015 (11) | Retrospective case series on 11 patients diagnosed with functional tics | London, UK | January 2011 to October 2013 | 11 | 37.5 (16-65) at onset  43.5 (19-66) at assessment | 27.3% F | Case review by  neurologist with  specialization in  functional  movement  disorders and tic  disorders | NR |  |
| Ducroizet et al., 2025 (12) | Prospective examination using structured interviews of children and adolescents diagnosed with FTLB during the pandemic | London, UK | January 2020 to March 2023 | 43 | 13 at onset  14.2 at assessment | 100% assigned female at birth  1 transgender man, 2 non-binary | NR | Information on comorbidities collected during follow-up interviews; parents and guardians reported previously received formal diagnoses |  |
| Firestone et al., 2023 (13) | Interviews with 8 students who were diagnosed with FTLB | Minnesota, USA | September 2021 to November 2021 | 8 | 16 (15-17) at onset | 100% F | NR | Standardized questionnaires including GAD-7 and PHQ-9 |  |
| Fremer et al., 2022 (14) | Clinical assessment of patients in a Tourette syndrome clinic who had mass social media-induced illness—functional Tourette-like behaviors | Hannover, Germany | May 2019 to September 2021 | 32 | 19.2 (10-53) at onset  20.1 (11-53) at assessment | 50% F | A neuropsychiatric evaluation by one clinician, who is a neurologist, psychiatrist, and TS expert | Clinical interview based on DSM-5 criteria by single evaluator, who is a neurologist, psychiatrist, and TS expert |  |
| Ganos et al., 2016 (15) | Cross-sectional review of patients who presented to a Tourette Syndrome clinic whose tics had “atypical features” | Hannover, Germany | 1995-2015 | 13 | 25.3 (5-50) at onset  31.0 (10-56) at evaluation | 30.8% F | NR | NR |  |
| Ganos et al., 2019 (16) | Cross-sectional review of patients with functional tics who presented to unspecified clinics | Not stated; both authors are affiliated with German universities | NR | 5 | 21.2 (7-49) at onset  32.2 (19-50) at evaluation | 40% F | NR | NR |  |
| Han et al., 2022 (17) | Retrospective review of all pediatric (age <18) patients referred to a tic clinic | Sydney, Australia | 2018 to July 2021 | 22 | NR (however, for the entire sample of 185 children, including 163 with CTD/TS, the mean age was 10.9 years) | 100% F | Part of routine clinical practice during initial clinical assessments | Part of routine clinical practice during initial clinical assessments |  |
| Larsh et al., 2022 (18) | Retrospective, cross-sectional review of patients with functional tics presenting to a pediatric movement disorders clinic | Cincinnati, USA | 2021 | 89 | 15.6 at evaluation | 93.2% F | NR | Parental report |  |
| Martino et al., 2023 (19) | Retrospective, cross-sectional review of patients with functional tics who were evaluated at one of ten different tertiary referral centers for tic disorders | Calgary, Canada; London, UK; Hannover, Germany; Sydney, Australia; Cincinnati, US; Catania, Italy; Paris, France; Budapest, Hungary; Strasbourg, France | September 2021 to June 2022 | 294 | 15.1 (8-53) at evaluation | 86.7% F | Diagnosis by tic disorder specialist according to criteria: “first onset or clinically relevant worsening starting since the last quarter of 2019; rapid escalation of severity of related impairment (in most cases within 4–8 weeks) and/or very early onset of complex tic-like behaviours during their evolution.” | Meeting DSM-5 criteria for each disorder by medical record review |  |
| Mathew et al., 2023 (20) | Retrospective, cross sectional review of pediatric patients diagnosed with functional tics at a single center | Charlottesville, USA | March 2020 to October 2021 | 29 | 15.8 at evaluation | 89.7% F | Determined by a pediatric neurologist with secondary review by a pediatric movement disorders specialist | EMR review |  |
| Maxwell et al., 2023 (21) | Retrospective, longitudinal study examining outcomes of intervention for functional tics | Australia | 2019 to 2023 | 8 | 14.6 (13-20) at onset | 100% F | Diagnosis made by clinical psychologist, psychiatrist, or pediatrician using ESSTS criteria | Unclear, but likely by clinical assessment by the same clinician who made the functional tic disorder diagnosis |  |
| Müller-Vahl et al., 2024 (22) | Retrospective data analysis of patients with comorbid Tourette Syndrome and functional tics | Hannover, Germany | 2002 to 2021 | 71 | 20.8 (5-52) at onset  21.5 (11-55) at evaluation | 38% F | Diagnoses of TS + FND were established by the lead study author (a neurologist) through chart review | Psychologist review of records |  |
| Nilles et al., 2024 (23) | Prospective evaluation of patients 6 and 12 months after their 1^st^ clinical visit; patients from clinical tic disorder registries | Calgary, Canada | October 2020 to December 2022 | 83  (baseline) | 18 (11-53) at evaluation | 95% F assigned at birth  23% trans or gender diverse | Clinically diagnosed by a movement disorder specialist | Age-appropriate questionnaires for ADHD, OCD, GAD, and MDD and were clinically diagnosed based on confirmatory psychiatric interviews using DSM-5 criteria. Diagnosis of autism was based on historical report. |  |
| Okkels et al., 2023 (24) | Retrospective review of medical records in pediatric patients diagnosed with functional tics | Herlev, Denmark | May 2020 to June 2021 | 28 | 14.4 (10.9-18.3) at onset  14.7 (11-18.9) at evaluation | 96.4% F | Examined by experienced neuropediatricians; physical neurological examination; multidisciplinary team and consensus | NR |  |
| Owen et al., 2022 (25) | Case presentation of 10 children who were diagnosed with functional tics at a specialty tic clinic | London, UK | January 2019 to January 2022 | 10 | 12.3 (9 to 14) at onset | 100% F | Diagnosed by a multi-disciplinary team | NR |  |
| Paulus et al., 2021 (26) | Cross-sectional comparison of patients with tic-like behaviors and Tourette Syndrome | Lubeck, Germany | NR | 13 | 15.31 at onset  16.54 (12-24) at evaluation | 38.5% F | NR | Clinical judgment | Only patients whose tics started after seeing a tic influencer on YouTube were included |
| Pringsheim et al., 2021 (27) | Prospective cohort study; registry enrolls participants at their ﬁrst clinic visit | Calgary, Canada | 2012 to June 2021 | 20 | 13.9 (13.1-14.7) at onset  14.3 (13.5-15) at evaluation | 95% F | Diagnoses performed by movement disorders specialists with expertise in tic disorders | Rating scales and questionnaires |  |
| Rigas et al., 2023 (28) | Case-based two-part survey; 8 experts asked to study 24 case videos | Berlin, Germany | NR | 24 | 26.7 at evaluation | 41.6% F | Expert diagnosis | NR |  |
| Robinson & Hedderly, 2016 (29) | Patients presenting with “tic attacks” (thought involve functional tics) to the Tic and Neurodevelopmental Movements service | London, UK | January 2014 to December 2015 | 12 | 11.25 (7.92- 15) at onset | 25% F | Multidisciplinary team using DSM-5 | Diagnosis made on the basis of DSM-5 criteria or by other professionals |  |
| Tomczak et al., 2024 (30) | Retrospective review of medical records of patients seen at Boston Children’s Hospital | Boston, USA | Beginning in March 2020 | 56 | 14 (10-18) at onset | 96% F | NR | Review of clinic notes |  |
| Trau et al., 2022 (31) | Retrospective chart review of consecutive children presenting to the UNC Clinic for Tourette Syndrome and Tic Disorders with a chief complaint of tics | Chapel Hill, USA | May 2020 to December 2021 | 31 | 14 at onset | 97% F | In-person evaluation; criteria defined by authors in paper similar to ESSTS includes age of onset, rapid onset, patient characteristics, and phenomenology | In-person evaluation; method not reported |  |

**Legend**: Abbreviations: ADHD, attention deficit hyperactivity disorder; DSM-5, Diagnostic and Statistical Manual of Mental Disorders; ESSTS, European Society for the Study of Tourette Syndrome; F, female; FT, functional tics; FTLB, functional tic-like behavior; GAD, generalized anxiety disorder; ICD, International Classification of Disease; MDD, major depressive disorder; MINI, Mini International Neuropsychiatry Interview; NR, not reported; OCB/OCD, obsessive compulsive behavior/obsessive compulsive disorder; PHQ-9, Patient Health Questionaire-9; STD, secondary tic disorder; TS, Tourette Syndrome; UK, United Kingdom; USA, United States of America

**References:**

1. Andersen K, Jensen I, Okkels KB, Skov L, Debes NM. Clarifying the Differences between Patients with Organic Tics and Functional Tic-Like Behaviors. HEALTHCARE. 2023 May 19;11(10).

2. Armstrong-Javors A, Realbuto E, Dy-Hollins ME, Scharf JM. Increase in Functional Tic Presentations in Sexual Orientation and Gender Identity Minority Youth During Coronavirus Disease 2019. Pediatr Neurol. 2024;155:182–6.

3. Baizabal-Carvallo JF, Alonso-Juarez M, Jankovic J. Contrasting features between Tourette syndrome and secondary tic disorders. J Neural Transm. 2023;130(7):931–6.

4. Berg L, Martino D, L’Erario ZP, Pringsheim T. Symptom Severity and Health Impacts of Functional Tic-Like Behaviors in Youth. Pediatr Neurol. 2024;155:68–75.

5. Burn O, Duncan M, McAllister E, Murphy T, Loewenberger A. The journey to a functional tics diagnosis and experiences of post diagnostic support: perspectives from adolescents and their parents. Disabil Rehabil. 2025;1–12.

6. Buts S, Duncan M, Owen T, Martino D, Pringsheim T, Byrne S, et al. Paediatric tic-like presentations during the COVID-19 pandemic. Arch Dis Child [Internet]. 2022;107(3). Available from: https://www.embase.com/search/results?subaction=viewrecord&id=L2021414872&from=export http://dx.doi.org/10.1136/ARCHDISCHILD-2021-323002

7. Cavanna AE, Caimi V, Capriolo E, Marinoni M, Arienti G, Riva A, et al. Neurodevelopmental Tics with Co-Morbid Functional Tic-like Behaviors: Diagnostic Challenges of a Complex Tourette Syndrome Phenotype. BRAIN Sci. 2025 Apr 23;15(5).

8. Cavanna AE, Purpura G, Riva A, Nacinovich R, Seri S. Functional tics: Expanding the phenotypes of functional movement disorders? Eur J Neurol. 2023;30(10):3353–6.

9. Cavanna AE, Purpura G, Riva A, Nacinovich R, Seri S. Neurodevelopmental versus functional tics: A controlled study. J Neurol Sci [Internet]. 2023;451. Available from: https://www.embase.com/search/results?subaction=viewrecord&id=L2025560618&from=export http://dx.doi.org/10.1016/j.jns.2023.120725

10. Cavanna AE, Purpura G, Riva A, Nacinovich R, Seri S. New-onset functional tics during the COVID-19 pandemic: Clinical characteristics of 105 cases from a single centre. Eur J Neurol. 2023;30(8):2411–7.

11. Demartini B, Ricciardi L, Parees I, Ganos C, Bhatia KP, Edwards MJ. A positive diagnosis of functional (psychogenic) tics. Eur J Neurol. 2015;22(3):527-e36.

12. Ducroizet A, Eccles C, Lancaster R, Kowalczyk A, Owen T, Sopena S, et al. Outcomes of functional tics in adolescents: A single-centre tertiary study. Arch Dis Child. 2025;110(7):528–32.

13. Firestone MJ, Holzbauer S, Conelea C, Danila R, Smith K, Bitsko RH, et al. Rapid onset of functional tic-like behaviors among adolescent girls-Minnesota, September-November 2021. Front Neurol. 2023/02/07 ed. 2022;13:1063261.

14. Fremer C, Szejko N, Pisarenko A, Haas M, Laudenbach L, Wegener C, et al. Mass social media-induced illness presenting with Tourette-like behavior. Front Psychiatry. 2022 Sept 20;13:963769.

15. Ganos C, Edwards MJ, Müller-Vahl K. “I swear it is Tourette’s!”: On functional coprolalia and other tic-like vocalizations. Psychiatry Res. 2016;246:821–6.

16. Ganos C, Müller-Vahl K. Cannabinoids in functional tic-like movements. Park Relat Disord. 2018/10/17 ed. 2019 Mar;60:179–81.

17. Han VX, Kozlowska K, Kothur K, Lorentzos M, Wong WK, Mohammad SS, et al. Rapid onset functional tic-like behaviours in children and adolescents during COVID-19: Clinical features, assessment and biopsychosocial treatment approach. J Paediatr Child Health. 2022;58(7):1181–7.

18. Larsh TR, Wu SW, Gilbert DL. Comparison of Impairment in Functional Tic Disorders Versus Tourette Syndrome. Pediatr Neurol. 2022;134:83–4.

19. Martino D, Hedderly T, Murphy T, Müller-Vahl KR, Dale RC, Gilbert DL, et al. The spectrum of functional tic-like behaviours: Data from an international registry. Eur J Neurol. 2023;30(2):334–43.

20. Mathew A, Abu Libdeh A, Patrie J, Garris J. Outcome in Pediatric Functional Tic Disorders Diagnosed During the COVID-19 Pandemic. J NEUROPSYCHIATRY Clin Neurosci. 2023;35(4):393–7.

21. Maxwell A, Zouki JJ, Eapen V. Integrated cognitive behavioral intervention for functional tics (I-CBiT): case reports and treatment formulation. Front Pediatr [Internet]. 2023;11. Available from: https://www.embase.com/search/results?subaction=viewrecord&id=L2026897057&from=export http://dx.doi.org/10.3389/fped.2023.1265123

22. Mueller-Vahl KR, Pisarenko A, Fremer C, Haas M, Jakubovski E, Szejko N. Functional Tic-Like Behaviors: A Common Comorbidity in Patients with Tourette Syndrome. Mov Disord Clin Pract. 2024 Mar;11(3):227–37.

23. Nilles C, Szejko N, Martino D, Pringsheim T. Prospective follow‐up study of youth and adults with onset of functional tic‐like behaviours during the COVID‐19 pandemic. Eur J Neurol. 2024 Jan 1;31(1):8.

24. Okkels KB, Skov L, Klanso S, Aaslet L, Grejsen J, Reenberg A, et al. Increased Number of Functional Tics Seen in Danish Adolescents during the COVID-19 Pandemic. Neuropediatrics. 2023;54(2):113–9.

25. Owen T, Silva J, Grose C, Bailey A, Robinson S, Anderson S, et al. Case report: Advice for schools on managing functional tic-like behaviours. Front Psychiatry [Internet]. 2022;13. Available from: https://www.embase.com/search/results?subaction=viewrecord&id=L2020657457&from=export http://dx.doi.org/10.3389/fpsyt.2022.1001459

26. Paulus T, Bäumer T, Verrel J, Weissbach A, Roessner V, Beste C, et al. Pandemic Tic-like Behaviors Following Social Media Consumption. Mov Disord. 2021;36(12):2932–5.

27. Pringsheim T, Ganos C, McGuire JF, Hedderly T, Woods D, Gilbert DL, et al. Rapid Onset Functional Tic-Like Behaviors in Young Females During the COVID-19 Pandemic. Mov Disord. 2021/08/14 ed. 2021 Dec;36(12):2707–13.

28. Rigas A, Mainka T, Pringsheim T, Münchau A, Malaty I, Worbe Y, et al. Distinguishing functional from primary tics: A study of expert video assessments. J Neurol Neurosurg Psychiatry. 2023;94(9):751–6.

29. Robinson S, Hedderly T. Novel Psychological Formulation and Treatment of “Tic Attacks” in Tourette Syndrome. Front Pediatr. 2016/06/01 ed. 2016;4:46.

30. Tomczak KK, Worhach J, Rich M, Swearingen Ludolph O, Eppling S, Sideridis G, et al. Time is ticking for TikTok tics: A retrospective follow-up study in the post-COVID-19 isolation era. Brain Behav [Internet]. 2024;14(3). Available from: https://www.embase.com/search/results?subaction=viewrecord&id=L2028941612&from=export http://dx.doi.org/10.1002/brb3.3451

31. Trau SP, Quehl L, Tsujimoto THM, Lin FC, Singer HS. Creating a Patient-Based Diagnostic Checklist for Functional Tics during the COVID-19 Pandemic. Neurol Clin Pract. 2022;12(5):365–76.
