# Supplementary material for: Psychiatric comorbidity in functional tics: a scoping review
Source: BMC Psychiatry. 2026 Mar 7;26:314. doi: 10.1186/s12888-026-07932-2 (PMC13081296; doi:10.1186/s12888-026-07932-2)
Supplement: Supplementary file 3 — Supplementary Material 3 [file 12888_2026_7932_MOESM3_ESM.docx]

**Additional File 3**

**Table 3. Prevalence of Comorbidities in Included Studies**

| **Author and Year** | **% with Depression** | **% with Anxiety** | **% with ADHD** | **% with OCD/OCB** | **% with Primary Tic Disorder** | **% with ASD** | **% with Other Functional Disorder or Symptoms** | **Other Psychiatric Disorders** | **Other Neurologic Disorders** | **Notes** |
| --- | --- | --- | --- | --- | --- | --- | --- | --- | --- | --- |
| Anderson et al., 2023 (1) | NR | 26.4% | 18.9% | 28.3% with OCD | NR | 13.2% | NR | 28.3% with other psychiatric diagnoses | NR | 79.2% with psychosocial trigger before/at symptom onset |
| Armstrong-Javors et al., 2024 (2) | NR | NR | 47% | 42% with OCD | 21% | NR | 37% | NR | NR |  |
| Baizabal-Carvallo et al., 2023 (3) | NR | NR | 14.28% | 23.8% with OCD | NR | NR | NR | NR | NR |  |
| Berg et al., 2024 (4) | 43.8% with current MDD | 37.5 with GAD | NR | 21.9% with OCD | NR | NR | NR | 9.4% with current bipolar 1; 42.8% with panic disorder; 43.8% with agoraphobia; 37.5% with social anxiety disorder; 18% with PTSD; 12.5% with SUD; 15.6% with current psychotic disorder; 6.3% with anorexia; 45.5% with borderline personality disorder | NR | 85.7% felt overwhelmed during pandemic |
| Burn et al., 2025 (5) | 28.6% | 42.9% with anxiety or GAD | 14.3% | 14.3% with OCD | 57.1% | 57.1% | 42.9% | 28.6% with PTSD | NR |  |
| Buts et al., 2022 (6) | 24% | Reported in 68%, diagnosed clinically in 50% | 57% | NR | NR | 50% | NR | NR | NR | For ADHD and ASD, comorbidity rates only reported for 14/34 patients seen in one specific clinic; 77% had seen videos of tics on social media |
| Cavanna et al., 2025 (7) | NR | 71.4% | 46% | 20.6% with OCD, 73% with OCB | 100% | 30.2% | 30.2% with functional seizures; 22.2% with other functional movement disorder | 52.4% with affective disorder | NR | All patients in this study had a TS diagnosis prior to FTLB diagnosis. |
| Cavanna et al., 2023 (8) | NR | 69.7% | NR | NR | NR | NR | 33.3% with functional seizures | 30.3% with affective disorder | NR |  |
| Cavanna et al., 2023 (9) | NR | 69.9% | 10.8% | 3.6% with OCD, 7.2% with OCB | NR | 25.3% | 47% with other FND; 37.3% with functional seizures; 20.5% with other functional movement disorders | 38.6% with affective disorder | NR |  |
| Cavanna et al., 2023 (10) | NR | 69.5% | 18.1% | 9.5% with OCD, 22.5% with OCB | 22.9% | 26.7% | 41% with other FND; 32.4% with functional seizures; 21% with other functional movement disorder | 40% | NR |  |
| Demartini et al., 2015 (11) | 45.5% | 27.2% | NR | NR | NR | NR | 72.7% | NR | NR | 91% had identified precipitating event |
| Ducroizet et al., 2025 (12) | 12% | 67% with GAD | 9% | 12% with OCD | NR | 19% | NR | 9% with social anxiety disorder; 2% with panic disorder; 2% with separation anxiety disorder | NR |  |
| Firestone et al., 2023 (13) | 100% with previous diagnosis of depression or current depression on screening | 100% with previous diagnosis of anxiety or current anxiety on screening | 25% | 12.5% | 0% | NR | NR | NR | NR | 38% watched videos of tics prior to onset |
| Fremer et al., 2022 (14) | 31.3% | 40.6% | 9.4% | 46.9% with OCB | 46.9% | 15.6% | 6.3% with pre-existing FTB | 81.3% with abnormal social behavior; 18.8% with personality disorder; 25.0% with sleeping problems; 15.6% with suicidal ideation; 9.4% with intellectual disability and comorbid conduct disorder, 6.3% with PTSD | NR | The counts for each of these comorbidities are different between the main manuscript text and supplemental table 2. Here, we tabulate the values from the manuscript text. |
| Ganos et al., 2016 (15) | NR | NR | 30.8% | 7.7% | 100% | NR | NR | NR | NR |  |
| Ganos et al., 2019 (16) | NR | NR | 60.0% | NR | 100% | NR | NR | 20.0% with borderline personality disorder; 20% with addiction to benzodiazepine | 20.0% with restless leg syndrome |  |
| Han et al., 2022 (17) | NR (see note) | NR (see note) | 13.7% | 22.7% with OCD | 0% | 9.1% | NR | NR | NR | 95.5% with “anxiety/depression” |
| Larsh et al., 2022 (18) | NR | NR | 50.0% (“ADHD, attention span, hyperactive/impulsive/disruptive behavior”) | 74.4% (“obsessive behavior or anxiety”); 29.8% (“compulsive behaviors or routines”) | NR | NR | NR | NR | NR | Reported values represent *parental concerns* for each set of symptoms. |
| Martino et al., 2023 (19) | 28% | 66% | 23% | 9% with OCD | 19% | 24% | NR | 37% with emotional traumatic experiences prior to FTLB onset; 17% with adverse experiences dating back to childhood; 6% with PTSD; 27% with recent or past suicidal behaviors; 10% with prior suicide attempts; 17% with suicidal ideation without prior suicide attempts | NR |  |
| Mathew et al., 2023 (20) | NR | 65.5% | 10.3% | NR | NR | NR | 34.5% with additional functional symptoms | 69.0% with mood disorder; 10.3% reported trauma history | NR |  |
| Maxwell et al., 2023 (21) | 37.5% | 100% | 12.5% (undiagnosed) | 50% with OCB | NR | 25% | 25.0% with functional seizures; 25% with functional numbness | 37.5% with alexithymia | NR |  |
| Müller-Vahl et al., 2024 (22) | 32% | 27% | 37% | 24% (OCD); 58% (OCS) | 100% | 3% | 34% with further medically unexplained symptoms, including fluctuating inability to walk or to speak, visual problems, paralysis, PNES, sudden temporary attacks of tremor or spasms, abdominal pain, dyspnea, and hyperventilation | 56.3% with self-injurious behaviors | NR | Mean (SD) number of psychiatric comorbidities: 2.3 (1.32) |
| Nilles et al., 2024 (23) | 42% with MDD | 65% with GAD | 35% | 7% with OCD | 100% | 4% | 30% developed other functional neurological symptoms concurrently or during follow-up period; 22.2% with functional seizures or syncopal attacks; 12.7% with functional motor symptoms | NR | NR |  |
| Okkels et al., 2023 (24) | 35.3% | 35.3% | 41.2% | 47.1% with OCD | ~11% | ~11% | NR | ~6% with PTSD | NR |  |
| Owen et al., 2022 (25) | 10% | 70% | 20% | 10% | 30% | 10% | 40% with non-epileptic seizures | 10% with compulsions;  10% with trauma;  20% with self-harm |  |  |
| Paulus et al., 2021 (26) | NR | NR | 15.4% | 30.8% | NR | 0% | NR | NR | NR |  |
| Pringsheim et al., 2021 (27) | 55% | 75% | 25% | 5% | NR | 0% | NR | NR | NR |  |
| Rigas et al., 2023 (28) | 37.5% | 20.8% | 29.2% | 25% | 66.7% | 4.2% | 4.2% with functional neurological disorder; 4.2% with functional seizures | 4.2% with panic attacks; 12.5% with borderline personality disorder, 8.3% with PTSD; 8.3% with eating disorder; 4.2% with cannabis addiction; 4.2% with alcohol addiction; 4.2% with alcohol and other addictions; 4.2% with oppositional defiant disorder | 4.2% with seizures; 4.2% with migraine |  |
| Robinson & Hedderly, 2016 (29) | 16.7% | 100% anxiety; 50% social anxiety disorder | NR | 33.3% | NR | 8.3% | NR | 8.3% with pica; 8.3% with specific phobias | 16.7% with frequent headaches |  |
| Tomczak et al., 2024 (30) | 71% | 93% | 45% | 23% | 32% prior history of tics | 7% | 32% with functional seizures; 7% with functional tremor | 23% with dissociative events | 13% with paralysis or gait impairment |  |
| Trau et al., 2022 (31) | NR | 90% | 68% | 58% | NR | NR | 16% with other functional neurological symptoms | NR | NR |  |

**Legend**: Abbreviations: ADHD, attention deficit hyperactivity disorder; DSM-5, Diagnostic and Statistical Manual of Mental Disorders; ESSTS, European Society for the Study of Tourette Syndrome; F, female; FT, functional tics; FTLB, functional tic-like behavior; GAD, generalized anxiety disorder; ICD, International Classification of Disease; MDD, major depressive disorder; NR, not reported; OCB/OCD, obsessive compulsive behavior/obsessive compulsive disorder; STD, secondary tic disorder; TS, Tourette Syndrome; UK, United Kingdom; USA, United States of America

**References:**

1. Andersen K, Jensen I, Okkels KB, Skov L, Debes NM. Clarifying the Differences between Patients with Organic Tics and Functional Tic-Like Behaviors. HEALTHCARE. 2023 May 19;11(10).

2. Armstrong-Javors A, Realbuto E, Dy-Hollins ME, Scharf JM. Increase in Functional Tic Presentations in Sexual Orientation and Gender Identity Minority Youth During Coronavirus Disease 2019. Pediatr Neurol. 2024;155:182–6.

3. Baizabal-Carvallo JF, Alonso-Juarez M, Jankovic J. Contrasting features between Tourette syndrome and secondary tic disorders. J Neural Transm. 2023;130(7):931–6.

4. Berg L, Pringsheim TM, Lerario M, Martino D. Psychological Factors Associated with Functional Tic-like Behaviours during the COVID-19 Pandemic. Res Child Adolesc Psychopathol. 2024;52(7):1157–72.

5. Burn O, Duncan M, McAllister E, Murphy T, Loewenberger A. The journey to a functional tics diagnosis and experiences of post diagnostic support: perspectives from adolescents and their parents. Disabil Rehabil. 2025;1–12.

6. Buts S, Duncan M, Owen T, Martino D, Pringsheim T, Byrne S, et al. Paediatric tic-like presentations during the COVID-19 pandemic. Arch Dis Child [Internet]. 2022;107(3). Available from: https://www.embase.com/search/results?subaction=viewrecord&id=L2021414872&from=export http://dx.doi.org/10.1136/ARCHDISCHILD-2021-323002

7. Cavanna AE, Caimi V, Capriolo E, Marinoni M, Arienti G, Riva A, et al. Neurodevelopmental Tics with Co-Morbid Functional Tic-like Behaviors: Diagnostic Challenges of a Complex Tourette Syndrome Phenotype. BRAIN Sci. 2025 Apr 23;15(5).

8. Cavanna AE, Purpura G, Riva A, Nacinovich R, Seri S. Functional tics: Expanding the phenotypes of functional movement disorders? Eur J Neurol. 2023;30(10):3353–6.

9. Cavanna AE, Purpura G, Riva A, Nacinovich R, Seri S. Neurodevelopmental versus functional tics: A controlled study. J Neurol Sci [Internet]. 2023;451. Available from: https://www.embase.com/search/results?subaction=viewrecord&id=L2025560618&from=export http://dx.doi.org/10.1016/j.jns.2023.120725

10. Cavanna AE, Purpura G, Riva A, Nacinovich R, Seri S. New-onset functional tics during the COVID-19 pandemic: Clinical characteristics of 105 cases from a single centre. Eur J Neurol. 2023;30(8):2411–7.

11. Demartini B, Ricciardi L, Parees I, Ganos C, Bhatia KP, Edwards MJ. A positive diagnosis of functional (psychogenic) tics. Eur J Neurol. 2015;22(3):527-e36.

12. Ducroizet A, Eccles C, Lancaster R, Kowalczyk A, Owen T, Sopena S, et al. Outcomes of functional tics in adolescents: A single-centre tertiary study. Arch Dis Child. 2025;110(7):528–32.

13. Firestone MJ, Holzbauer S, Conelea C, Danila R, Smith K, Bitsko RH, et al. Rapid onset of functional tic-like behaviors among adolescent girls-Minnesota, September-November 2021. Front Neurol. 2023/02/07 ed. 2022;13:1063261.

14. Fremer C, Szejko N, Pisarenko A, Haas M, Laudenbach L, Wegener C, et al. Mass social media-induced illness presenting with Tourette-like behavior. Front Psychiatry [Internet]. 2022;13. Available from: https://www.embase.com/search/results?subaction=viewrecord&id=L2019406628&from=export http://dx.doi.org/10.3389/fpsyt.2022.963769

15. Ganos C, Edwards MJ, Müller-Vahl K. “I swear it is Tourette’s!”: On functional coprolalia and other tic-like vocalizations. Psychiatry Res. 2016;246:821–6.

16. Ganos C, Müller-Vahl K. Cannabinoids in functional tic-like movements. Park Relat Disord. 2018/10/17 ed. 2019 Mar;60:179–81.

17. Han VX, Kozlowska K, Kothur K, Lorentzos M, Wong WK, Mohammad SS, et al. Rapid onset functional tic-like behaviours in children and adolescents during COVID-19: Clinical features, assessment and biopsychosocial treatment approach. J Paediatr Child Health. 2022;58(7):1181–7.

18. Larsh TR, Wu SW, Gilbert DL. Comparison of Impairment in Functional Tic Disorders Versus Tourette Syndrome. Pediatr Neurol. 2022;134:83–4.

19. Martino D, Hedderly T, Murphy T, Müller-Vahl KR, Dale RC, Gilbert DL, et al. The spectrum of functional tic-like behaviours: Data from an international registry. Eur J Neurol. 2023;30(2):334–43.

20. Mathew A, Abu Libdeh A, Patrie J, Garris J. Outcome in Pediatric Functional Tic Disorders Diagnosed During the COVID-19 Pandemic. J NEUROPSYCHIATRY Clin Neurosci. 2023;35(4):393–7.

21. Maxwell A, Zouki JJ, Eapen V. Integrated cognitive behavioral intervention for functional tics (I-CBiT): case reports and treatment formulation. Front Pediatr [Internet]. 2023;11. Available from: https://www.embase.com/search/results?subaction=viewrecord&id=L2026897057&from=export http://dx.doi.org/10.3389/fped.2023.1265123

22. Mueller-Vahl KR, Pisarenko A, Fremer C, Haas M, Jakubovski E, Szejko N. Functional Tic-Like Behaviors: A Common Comorbidity in Patients with Tourette Syndrome. Mov Disord Clin Pract. 2024 Mar;11(3):227–37.

23. Nilles C, Szejko N, Martino D, Pringsheim T. Prospective follow-up study of youth and adults with onset of functional tic-like behaviours during the COVID-19 pandemic. Eur J Neurol. 2023/08/30 ed. 2024 Jan;31(1):e16051.

24. Okkels KB, Skov L, Klanso S, Aaslet L, Grejsen J, Reenberg A, et al. Increased Number of Functional Tics Seen in Danish Adolescents during the COVID-19 Pandemic. NEUROPEDIATRICS. 2023 Apr;54(02):113–9.

25. Owen T, Silva J, Grose C, Bailey A, Robinson S, Anderson S, et al. Case report: Advice for schools on managing functional tic-like behaviours. Front Psychiatry [Internet]. 2022;13. Available from: https://www.embase.com/search/results?subaction=viewrecord&id=L2020657457&from=export http://dx.doi.org/10.3389/fpsyt.2022.1001459

26. Paulus T, Bäumer T, Verrel J, Weissbach A, Roessner V, Beste C, et al. Pandemic Tic-like Behaviors Following Social Media Consumption. Mov Disord. 2021;36(12):2932–5.

27. Pringsheim T, Ganos C, McGuire JF, Hedderly T, Woods D, Gilbert DL, et al. Rapid Onset Functional Tic-Like Behaviors in Young Females During the COVID-19 Pandemic. Mov Disord. 2021/08/14 ed. 2021 Dec;36(12):2707–13.

28. Rigas A, Mainka T, Pringsheim T, Münchau A, Malaty I, Worbe Y, et al. Distinguishing functional from primary tics: A study of expert video assessments. J Neurol Neurosurg Psychiatry. 2023;94(9):751–6.

29. Robinson S, Hedderly T. Novel Psychological Formulation and Treatment of “Tic Attacks” in Tourette Syndrome. Front Pediatr. 2016 May 11;4.

30. Tomczak KK, Worhach J, Rich M, Swearingen Ludolph O, Eppling S, Sideridis G, et al. Time is ticking for TikTok tics: A retrospective follow-up study in the post-COVID-19 isolation era. Brain Behav. 2024/03/12 ed. 2024 Mar;14(3):e3451.

31. Trau SP, Quehl L, Tsujimoto THM, Lin FC, Singer HS. Creating a Patient-Based Diagnostic Checklist for Functional Tics during the COVID-19 Pandemic. Neurol Clin Pract. 2022;12(5):365–76.
